# Comparison of glide paths created with K-files, PathFiles, and the ProGlider file, and their effects on subsequent WaveOne preparation in curved canals

**DOI:** 10.1186/s12903-018-0614-3

**Published:** 2018-08-29

**Authors:** Linxia Zheng, Xiongfei Ji, Chengxi Li, Lulu Zuo, Xin Wei

**Affiliations:** 1 Jiangsu Key Laboratory of Oral Diseases, Nanjing, China; 2 Department of Conservative Dentistry and Endodontics, School of Stomatology, Nanjing Medical University, Han-Zhong Road 136th, Nanjing, 210029 Jiangsu China; 3 Now at Department of Conservative Dentistry and Endodontics, The Affiliated Stomatological Hospital of Suzhou University, Suzhou, China

**Keywords:** Apical extruded debris, Canal transportation, Canal volume, Glide path, ProGlider, PathFile, WaveOne, Working time

## Abstract

**Background:**

The root canal glide path has been recommended as the foundation for a safer root canal preparation. The aim of this study was to compare glide paths created with K-files, PathFiles, and the ProGlider file, and their effects on subsequent WaveOne preparation regarding canal transportation, canal volume increase, apical extruded debris, and working time in curved canals.

**Methods:**

Sixty mesial canals of extracted human mandibular first molars were randomly assigned to the K-file (KF), PathFile (PF) and ProGlider file (PG) groups for glide path preparation. Then, canals were prepared using WaveOne files. Specimens were scanned (voxel size: 18 μm) three times using micro-computed tomography: pre-glide path, post-glide path, and post-root canal preparation. Canal transportations were measured at 1, 3, and 5 mm levels from the apical foramen, and canal volume increases were also accounted. Apical extruded debris during preparation was collected for measurement. Meanwhile,working time was recorded. Data were analyzed statistically using one-way analysis of variance and Tukey’s post hoc tests (*p* < 0.05).

**Results:**

After glide path preparation, the PG and PF groups showed significantly less canal transportation than the KF group at all levels (*P* < 0.05), while the PG group exhibited a significantly larger canal volume increase than the PF and KF groups (*P* < 0.05). After the subsequent canal preparation with WaveOne, the PG and PF groups showed significantly less canal transportation than the KF group at 3 and 5 mm levels, and the PG group showed significantly less canal transportation than the PF group at 5 mm level (*P* < 0.05). However, statistically similar canal volume increases occurred among the three groups. Additionally, the PG and PF groups produced less apical extruded debris compared to the KF group (*P* < 0.05). The working time of the PG group was the shortest, while that of the KF group was the longest.

**Conclusion:**

Compared with the PathFiles and K-files, the ProGlider file combined with the WaveOne file showed reduced canal transportation and working time.

## Background

Endodontic root canal glide path has been recommended as the foundation for a safer root canal preparation [1, 2]. The glide path is defined as a smooth tunnel running from the canal orifice to the physiological apical foramen [2]. A successful glide path reduces torsional stress, creates a smooth and original shape for subsequent root canal preparation, and reduces procedural errors such as instrument failure, canal transportation and ledge formation [3–7].

The WaveOne single-file system (Dentsply Sirona, York, Pennsylvania) is a reciprocating system used in clinics to shape the root canal completely from start to finish. The WaveOne system has exhibited good bending and torsional resistance [8, 9]. However, some investigators have observed notable canal transportation in curved canals after WaveOne preparation and that the WaveOne file extruded more debris to the foramen, which negatively affects the prognosis of root canal treatment [10–12]. Apical extruded debris during root canal treatment is associated with postoperative pain, postoperative inflammation and even persistent apical periodontitis [13, 14]. In addition, instrument failure still occurs during single use of WaveOne. To improve the performance of the WaveOne file, clinicians create a glide path before the WaveOne preparation.

In the majority of cases in clinics, a manual glide path created with stainless steel hand K-files is a reliable technique [15]. However, creation of a glide path with hand files can be time-consuming and technique-sensitive and may result in poor preparation outcomes [3, 16, 17]. Hence, investigators have recently focused on using mechanical glide path files to achieve a safer and more predictable glide path. The PathFile multiple-file system (Dentsply Sirona) and the ProGlider single-file system (Dentsply Sirona) are two kinds of nickel titanium (NiTi) glide path file systems used in clinics. The PathFile system is manufactured out of conventional NiTi alloy, and consists of three instruments with a fixed 0.02 taper, ISO 13, 16 and 19 tip sizes, and a square cross section [3]. The ProGlider system is manufactured using a heat-treated M-Wire NiTi alloy, which enhances its flexibility and cyclic fatigue resistance [18–20], and consists of a single instrument with a progressive taper (0.02 at tip level up to 0.085), an ISO 16 tip size, and a square cross section [21]. It has been demonstrated experimentally that the creation of glide paths with the PathFiles and ProGlider file is faster and experiences fewer procedural errors than that with the K-files [3, 18–20].

Presently, there is no published study of the ProGlider file combined with the WaveOne file for a curved canal preparation. Hence, the aim of this study was to compare the glide paths created with the ProGlider file, PathFiles and K-files, and their effects on subsequent WaveOne shaping. The null hypothesis was that there would be no differences in canal transportation, canal volume increase, apical extruded debris, and working time among groups after the creation of glide paths using K-files, PathFiles and the ProGlider file and after the subsequent preparation using WaveOne file in curved root canals.

## Methods

### Specimen selection

In summary, after approval by the Ethical Committee Department of Dentistry Hospital, Nanjing Medical University (Institutional Review Board) (IRB; approval number PJ2015–001-08), human mandibular first molars freshly extracted for reasons unrelated to this research were collected and stored in distilled water at 4 °C before selection. Only fully developed teeth with two separate mesial roots ending in two fully formed apices were selected. Meanwhile, the teeth also had to have curvatures of 25°-40° according to Schneider’s [22] method and a maximum curvature located within the middle third of the root canal. A standard endodontic access cavity was prepared, and a number #08 stainless steel manual K-file (Dentsply Sirona) was pre-curved and inserted through the mesial canal to ensure apical patency. To increase standardization, the root canals that could be negotiated with a #10 K-file (or larger) up to the apex without any resistance were excluded, and the crown and distal root of each tooth were flattened. The working length (WL) was measured as 0.5 mm short of the length when the tip of the instrument was just observed at the apical foramen. After the WL measurement, canals with WL shorter than 11 mm were excluded, and canals with WL longer than 11 mm were standardized to 11 mm using a high-speed bur. All of the root selection procedures were performed by one endodontist. Accordingly, 60 mesiobuccal and mesiolingual canals of 39 teeth were selected and randomly assigned into 3 groups for 20 canals each. Then, the balance of canal curvatures among the three groups was analyzed and confirmed by one-way analysis of variance (*p* > 0.05).

### Canal preparation

The K-files(KF), PathFiles(PF), ProGlider file(PG), and WaveOne file(WO) were used for the canal preparation.

Group KF + WO: the glide path was prepared using pre-curved stainless steel K-files (15#, 0.02 and 20#, 0.02), and the root canal was subsequently prepared by WaveOne Primary (25#, 0.08) file.

Group PF + WO: the glide path was prepared using PathFiles (13#, 0.02, 16#, 0.02, 19#, 0.02), and the root canal was subsequently prepared by WaveOne Primary (25#, 0.08) file.

Group PG + WO: the glide path was prepared using the ProGlider single file (16#, 0.02 to 0.085), and the root canal was subsequently prepared by WaveOne Primary (25#, 0.08) file.

Both the PF and PG NiTi glide path files were applied with an endodontic motor (X-Smart plus, Dentsply Sirona) operated with a 16:1 contra angle, at 300 rpm and with 5 Ncm torque**.** The WaveOne file was performed to the WL with the WaveOne program using the same endodontic motor with a slow, in-and-out pecking motion according to the manufacturer’s instructions. After every three pecking motions, the WaveOne file was taken out of the canal and cleaned with gauze. The WL was checked using a #10 K-file, and the canal was irrigated with 10 mL distilled water using a syringe with a 30-gauge side-vented irrigation needle (Wode, Zhenjiang, China) as the file was taken out of the canal each time. Meanwhile, the instrument failure was recorded. To avoid inter-operator variability, the canal preparation was performed by a single experienced endodontist.

### Canal transportation and canal volume analysis

The high-resolution micro-computed tomography (micro-CT) scanner SkyScan 1176 (Bruker microCT, Kontich, Belgium) was used to record the canal transportation and canal volume increase. Each of the studied teeth was scanned for three times: before glide path preparation, after glide path preparation and after root canal preparation. The parameters were kept constant: 70 kV, 353 μA, a 0.5-mm-thick aluminum filter, 360° rotations and a 0.5° rotation step, displaying an object with an 18 μm voxel size. After the scanning, the images were reconstructed and the measurements were acquired using CTAn v1.10.1.0 software (Bruker microCT). Root canal transportations were analyzed at three levels: 1 mm, 3 mm and 5 mm from the apical foramen. The following formula developed by Gambill et al. [23] was used to measure canal transportation: │(m1-m2)-(d1-d2)│, where m1 indicates the thinnest mesial canal wall pre-instrumentation, m2 indicates the thinnest mesial canal wall post-instrumentation, d1 indicates the thinnest distal canal wall pre-instrumentation, and d2 indicates the thinnest distal canal wall post-instrumentation. The root canal volume was measured, then the volume increase was determined by subtracting the volume of the untreated canal from the volume of the treated canal. All of the data were measured and analyzed by an investigator who was blinded to the specimen assignment.

### Debris collection and evaluation

The experimental equipment used to collect the apical extruded debris was similar to that described by Myers and Montgomery [24]. Each tooth was fixed on a stopper and then attached to a pre-weighted Eppendorf tube. A 25-gauge needle was used alongside the stopper to balance the air pressure inside and outside of the tube. Then, the tube was fitted into a vial. The apical extruded debris during the glide path preparation and the WaveOne preparation was collected into the tube, and the debris visually adherent to the external surface of the apex was collected into the tube by flushing the apex with 0.5 mL of distilled water. The tube was then stored in an incubator at 70 °C for 5 days to evaporate moisture before finally weighing the tube on a microbalance to 10^− 5^ g precision (AY 120 Analytic Balance, Shimadzu Corporation, Tokyo, Japan). Each tube was measured three times and the mean value was recorded. The net weight of the dry debris was determined by subtracting the original weight of the empty Eppendorf tube from the gross weight. The evaluation was performed by an experimenter who was blinded to group assignment.

### Working time

Working time was recorded with an electronic stopwatch, including total active instrumentation phase, cleaning of the flutes of the instruments, checking of the WL and irrigation, while the time required to adjust the rubber stops to the WL was not included.

### Statistical analysis

Normality of variable distribution was evaluated with the Kolmogorov-Smirnov test (SPSS 17.0 software; SPSS Inc., Chicago, IL, USA). Then, the data were statistically analyzed using a one-way analysis of variance. Multiple comparisons were made by using Tukey’s test. The level of significance was set at *P* < 0.05.

## Results

No instrument failure occurred during root canal preparation.

**Fig. 1:**
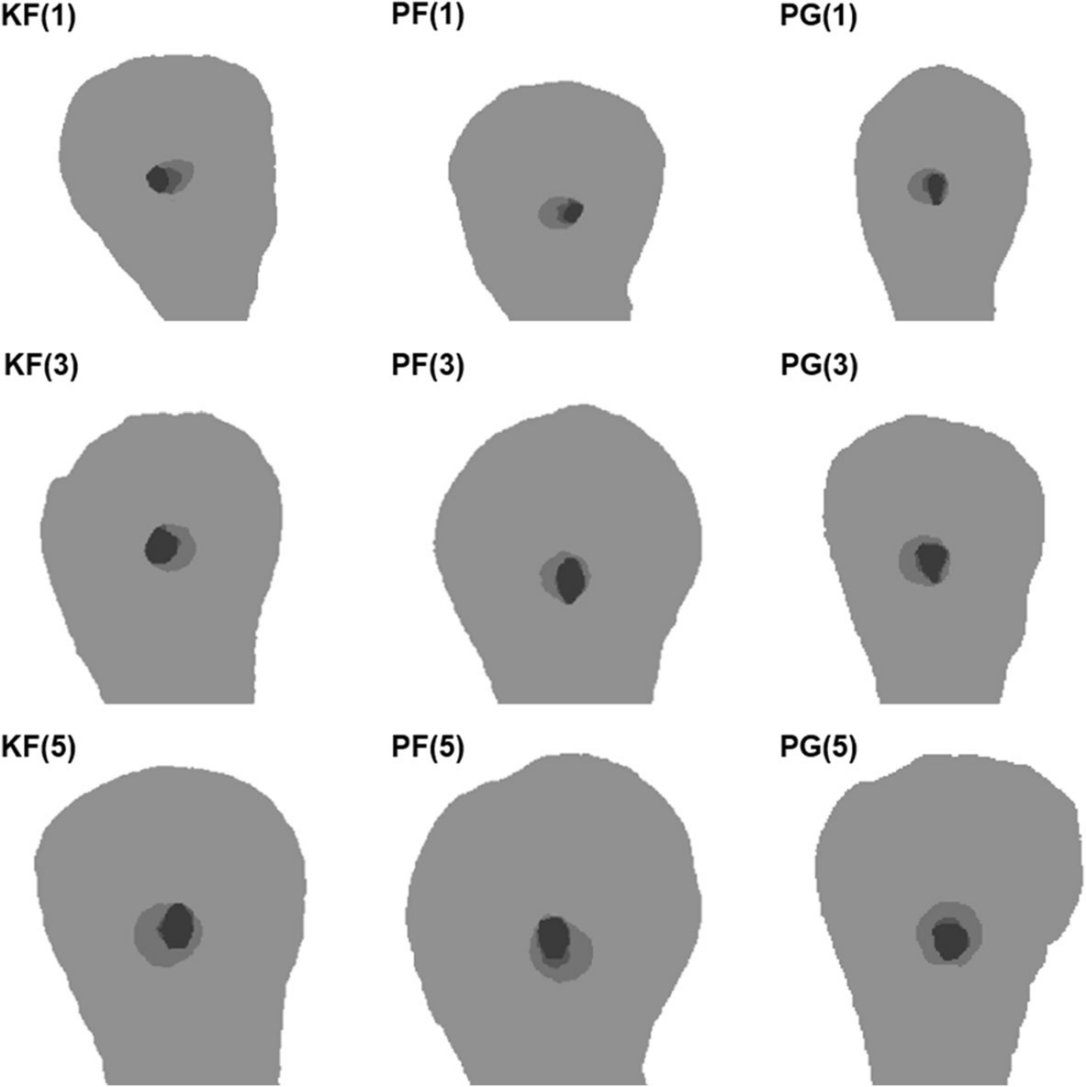
Image matching of pre-glide path preparation, post-glide path preparation and post-root canal preparation. Note the difference between pre-glide path (dark grey), post-glide path (neutral grey) and post-root canal preparation (light gray) at the 1, 3, and 5 mm levels from the apical foramen. KF: K-files; PF: PathFiles; PG: ProGlider file

After glide path preparation, the KF group showed significantly more canal transportation than both the PG and PF NiTi file groups at all levels (*P* < 0.05), while the two NiTi file groups displayed statistically similar canal transportation at all levels (*P* > 0.05). In addition, the canal volume increase was larger in the PG group compared to that in the PF and KF groups (*P* < 0.05) (Table 1; Fig. 1).

**Table 1:** Canal volume increase and canal transportation of K-files, PathFiles and the ProGlider file groups after glide path preparation (mean ± standard deviation)

Group	Volume increase(mm^3^)	Transportation(mm)		
		1 mm	3 mm	5 mm
KF	0.4771 ± 0.0987^a^	0.0280 ± 0.0041^a^	0.0205 ± 0.0023^a^	0.0117 ± 0.0033^a^
PF	0.4467 ± 0.1187^a^	0.0201 ± 0.0045^b^	0.0164 ± 0.0031^b^	0.0070 ± 0.0021^b^
PG	0.5530 ± 0.0710^b^	0.0181 ± 0.0036^b^	0.0148 ± 0.0027^b^	0.0056 ± 0.0022^b^

*KF* glide path with K-files, *PF* glide path with PathFiles, *PG* glide path with the ProGlider file. Different superscript letters in the same column indicate significant differences between groups (*P* < 0.05)

After the subsequent canal preparation using the WaveOne file, canal transportation at the 3 mm and 5 mm levels was significantly lower in the PG and PF groups than that in the KF group (*P* < 0.05), whereas no significant difference in canal transportation at the 1 mm level was found among the three groups (*P* > 0.05). Furthermore, the PG group displayed significantly less canal transportation than the PF and KF groups at the 5 mm level (*P* < 0.05). However, statistically similar canal volume increases were found among the three groups (*P* > 0.05) (Table 2, Fig. 1).

**Table 2:** Canal volume increase and canal transportation of K-files, PathFiles and the ProGlider file groups after subsequent WaveOne preparation (mean ± standard deviation)

Group	Volume increase(mm^3^)	Transportation(mm)		
		1 mm	3 mm	5 mm
KF + WO	2.9621 ± 0.6976^a^	0.2106 ± 0.0347^a^	0.1743 ± 0.0216^a^	0.0858 ± 0.0290^a^
PF + WO	2.9055 ± 0.6249^a^	0.1932 ± 0.0311^a^	0.1408 ± 0.0228^b^	0.0671 ± 0.0186^b^
PG + WO	2.8798 ± 0.5538^a^	0.1885 ± 0.0250^a^	0.1252 ± 0.0188^b^	0.0464 ± 0.0118^c^

KF + WO, K-files before WaveOne; PF + WO, PathFiles before WaveOne; PG + WO, the ProGlider file before WaveOne. Different superscript letters in the same column indicate significant differences between groups (*P* < 0.05)

Both the PG and PF NiTi glide path file groups produced significantly less apical extruded debris than the KF group (*P* < 0.05), while no significant difference was found between the two NiTi file groups (*P* > 0.05) (Table 3).

**Table 3:** Apical extruded debris and working time of K-files, PathFiles and the ProGlider file groups (mean ± standard deviation)

Group	KF + WO	PF + WO	PG + WO
Debris(g)	0.00144 ± 0.00045^a^	0.00049 ± 0.00022^b^	0.00043 ± 0.00017^b^
Time(s)	246.76 ± 31.80^a^	157.34 ± 22.54^b^	128.46 ± 19.21^c^

KF + WO, K-files before WaveOne; PF + WO, PathFiles before WaveOne; PG + WO, the ProGlider file before WaveOne. Different superscript letters in the same column indicate significant differences between groups (*P* < 0.05)

The working time analysis showed that the working time of the PG group was the shortest, while that of the KF group was the longest. (*P* < 0.05) (Table 3).

## Discussion

In the present study, curved canals of extracted human mandibular first molars were used as the specimens. Curved canals present greater challenges to instrumentation [17, 22], which have been associated with pointing up performance differences among various instrument systems [3, 25]. In addition, extracted human mandibular first molars provide natural dentin and are thus better experimental subjects. Although higher standard deviations are observed in natural canals than in artificial canals [16], natural canals are still commonly used in numerous studies. In this study, the equilibrium tests were used between the studied groups of the natural canal to balance the samples and reduce the standard deviations.

Canal transportation and volume increases were measured using a micro-CT scanner, a nondestructive technique capable of accurately reproducing internal and external tooth morphologies and accurately determining surface and volume changes after instrumentation [26]. Canal transportation was evaluated at 1, 3, and 5 mm levels from the apical foramen in this study, because these three levels are in the apical area where transportation potentially occurs [6, 27].

In the present study, after glide path preparation, the ProGlider file produced a significantly larger canal volume increase compared to the PathFiles and K-files. It is supposed that the progressive tapered design of the ProGlider file created a preliminary enlargement of the root canal at the coronal and middle portions. However, there was no significant difference in the root canal volume increase among the three groups after further WaveOne preparation. The results suggested that the subsequent WaveOne preparation eliminated the differences in canal volume increases caused by glide path preparation.

Post-glide path preparation analysis indicated less canal transportation in both the NiTi glide path file groups than in the stainless steel K-file group at all levels examined in this study. Several other studies have also found that NiTi glide path files produced less canal transportation than stainless steel K-files, resulting from the better flexibility of NiTi files [1, 16]. After further canal preparation with the WaveOne file, the ProGlider file group showed less overall canal transportation than the PathFile and K-file groups. This result might be explained by the coronal and middle portions enlargement of the root canal by the ProGlider file, which reduced the torsional stress for the subsequent WaveOne preparation. Berutti et al. found that after preliminary enlargement of the root canal using PathFiles, the WaveOne file more easily achieved a working length and produced less canal transportation [5]. Additionally, another report showed that coronal enlargement of the root canal using a ProTaper Universal SX reduced canal transportation during WaveOne preparation [12]. The findings of the present study are in agreement with these previous observations mentioned above. Furthermore, other reports have also shown that the ProGlider file reduced resistance and root canal transportation caused by the ProTaper Next system [18, 21, 28].

The WaveOne file applied with a reciprocating and in-and-out pecking motion may act as a piston and extrude more debris to the foramen [11], which may increase the incidence of the postoperative pain, postoperative inflammation and even persistent apical periodontitis [13, 14]. In this study, creation of glide paths with the ProGlider file and PathFiles reduced the amount of apical extruded debris better than that with K-files [7], which is good for decreasing the postoperative reactions caused by the WaveOne file. The reduced amount of debris in the NiTi glide path file groups may be explained by the reduced canal transportation elicited by the NiTi glide path files, which means that there is less unnecessary removal of normal dentin with the NiTi glide path files than with the hand K-files.

The high-efficiency of the ProGlider file when combined with the ProTaper Next system was demonstrated in previous study [28]. The working time results in this study showed that the combination of the ProGlider file and WaveOne file also achieved the highest efficiency in the canal preparation among the three groups. This may be because the single-file design of the ProGlider file distinctly reduced the working time of the glide path, which was previously reported [1]. On the other hand, enlargement of the coronal and middle portions by the ProGlider file may have reduced the torsional stress for the subsequent root canal preparation so that the WaveOne file could more easily achieve a working length.

## Conclusion

In conclusion, compared with the PathFiles and K-files,the ProGlider file combined with the subsequent WaveOne file showed reduced canal transportation and working time, and the ProGlider file and PathFiles showed reduced apical extruded debris compared to the K-files.

## Abbreviations

KF: K-file
NiTi: Nickel titanium
PF: PathFile
PG: ProGlider
WL: Working length
WO: WaveOne
